# Shelf-life, quality, safety evaluations of blueberry fruits coated with chitosan nano-material films

**DOI:** 10.1038/s41598-020-80056-z

**Published:** 2021-01-08

**Authors:** Yang Li, Sami Rokayya, Fuguo Jia, Xin Nie, Jingwen Xu, Abeer Elhakem, Manal Almatrafi, Nada Benajiba, Mahmoud Helal

**Affiliations:** 1 College of Food Science, Northeast Agricultural University, Harbin, 150030 Heilongjiang China; 2 Department of Food Science and Nutrition, College of Sciences, Taif University, P.O. 11099, Taif, 21944 Saudi Arabia; 3 College of Engineering, Northeast Agricultural University, Harbin, 150030 Heilongjiang China; 4 Department of Biology, College of Science and Humanities in Al-Kharj, Prince Sattam Bin Abdulaziz University, Al-Kharj, 11942 Saudi Arabia; 5 Department of Nutritional Sciences, University of Connecticut, Storrs, CT 06269 USA; 6 Department of Basic Health Sciences, Deanship of Preparatory Year, Princess Nourah Bint Abdulrahman University, P.O. Box 84428, Riyadh, 11671 Saudi Arabia; 7 Department of Mechanical Engineering, Faculty of Engineering, Taif University, P.O. 11099, Taif, 21944 Saudi Arabia; 8 Production and Mechanical Design Department, Faculty of Engineering, Mansoura University, Mansoura, 35516 Egypt

**Keywords:** Biochemistry, Biological techniques, Biophysics, Biotechnology, Cell biology, Chemical biology, Microbiology, Plant sciences, Engineering, Materials science, Nanoscience and technology

## Abstract

Chitosan coating (B/CH) in addition with nano-material films as silicon (B/CH/Nano-SiO_2_) and titanium (B/CH/Nano-TiO_2_) dioxides were developed and applied to detect potential changes on fresh blueberry fruits in commercial storage temperature. Physical, mechanical parameters (weight loss, decay rate, colour index and firmness), phytochemical contents (ascorbic acid, acidity, soluble solids concentration, titratable acidity, and repining index), phenolic enzymes (peroxidase and polyphenoloxidase), pigments (anthocyanin) and microbiological analysis (mesophilic aerobic, yeasts and molds populations) were detected every other day until the end of the experiment. Nano-coating based on (Nano-TiO_2_) established the most suitable values for weight loss (2.22%), titratable acidity (0.45% citric acid), and repining index. (B/CH/Nano-TiO_2_) reported a gradual increase in polyphenoloxidase and peroxidase enzyme activities (659.45 U/min g) and (20.39 U/min g), respectively. While, (B/CH/Nano-SiO_2_) established the slightest change in acidity (2.61), anthocyanin (105.19 cyanidin-3-*O*-glucoside mg/100 g FW) and minimized the growth of mesophilic aerobic, yeasts, and molds populations (3.73–3.98 log CFU/g), respectively. (B/CH) films maintained lightness (6.80% loss) and recorded the highest ascorbic acid content (7.34 g/100 g FW). Therefore, chitosan nano-material films can maintain nutrients and control the microbial growth for extending the shelf life of fresh blueberry fruits.

## Introduction

Blueberry fruit (*Vacciniumashei *L.) is one of the main potential functional food which can act as anticarcinogenic and has therapeutic properties due to the presence of bioactive components such as anthocyanin and phenolics^1^. Blueberries have plenty of essential nutrients such as vitamins (A, B, C, and K), minerals (P, Mg, Fe, Ca, K, Mn), and low levels of fats and sodium. Blueberries have an essential role in preventing bone loss, protecting the liver, atherosclerosis, anticarcinogenic, antihypertensive, hypoglycemic, and regulate the inflammatory responses^2^. Although blueberry fruit is considered as a very perishable fruit as the proper storage condition is approximately 4 °C, with a relative humidity of (90–95%) which costs the producers plenty of money and space to guarantee the fruit quality and avoid any sight of spoilage^3^. Several studies showed that edible coatings had improved the shelf-life and protect from the external environmental influences, such as chitosan, starch, pectin, alginate, pullulan, quinoa protein, and sunflower oil during room temperature^4,5^. In addition, other studies focused on the effect of chilling on blueberries preservation^6,7^. Various preservation technologies have been applied in fresh blueberries such as gamma irradiation, ozonation, chlorine oxidase, and some sanitizers (captan, difolatan, hexanal, and vinegar vapors)^8^. Recently, there is an increasing interest in nano-films applications or coatings in food technology to extend the shelf-life and produce low-pH fresh vegetables and fruits^9^. Nearly, half percent of the postharvest blueberries are inconsumable due to loss of texture, weight, quality deterioration, and/or microbial growth, especially due to molds and fungal outbreaks (e.g., *Botrytis cinerea*)^10^. Nano-films are defined as a very thin layer, less than 100 nm that can be used as a coating layer over the fruit or as an external film to control dehydration, shelf-life limitation, polyphenolics oxidation, transpiration, enzymes, respiration, exposure to oxygen and light, microbial attack to achieve the consumer acceptability^11^. Coatings can provide pre-washed or even ready-to-eat blueberries as new products in supermarkets^12^.

The aim of this research work was to describe the effects of chitosan, titanium, and silicon dioxide nanoparticles coating films (in-vitro and in-vivo) on fresh blueberries samples to evaluate the postharvest quality prolong shelf lifeduring commercial storage for outlook industrialized development.

## Results

### Weight loss and decay rate

**Figure 1:**
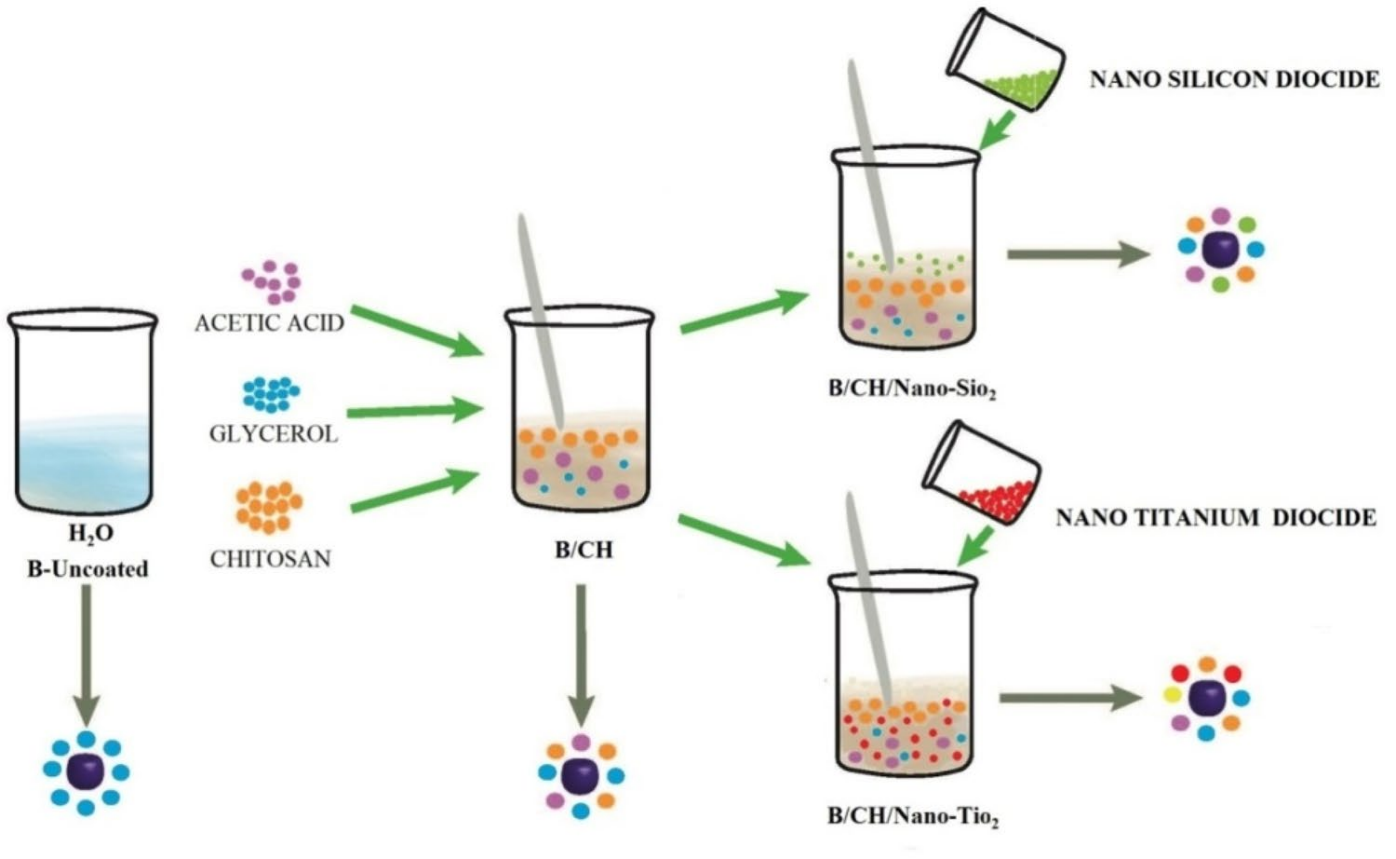
Films preparation.

**Figure 2:**
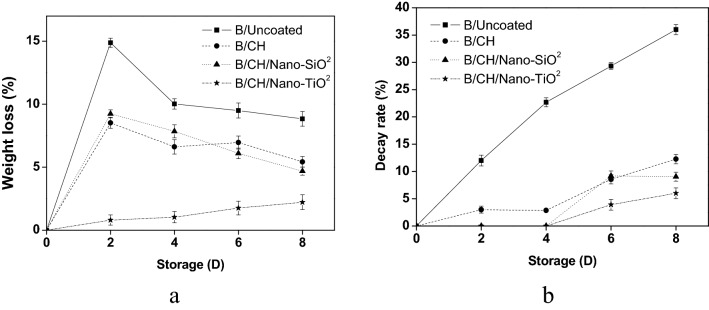
Effects of coating films on weight loss (**a**) and (**b**) decay rate.

In general, Fig. 1 shows the films preparations, there was high weight loss in all the coated blueberries after the whole storage period (Fig. 2a). B/CH/Nano-TiO_2_ coated treatment significantly established reduced weight loss (2.22%) compared to B/CH and B/CH/Nano-SiO_2_ coated samples (5.43 and 4.69%, respectively). Though, the maximum loss was achieved by B/Uncoated blueberry samples was (8.83%).

(B/CH/Nano-TiO_2_) followed by (B/CH/Nano-SiO_2_) coating treatments controlled decay rates (5.99–9.04%) on the 8th day at the ambient temperature, Fig. 2b. Karolina et al.^13^, established comparable results for fresh *Highbush* blueberry.

### Colour index and firmness texture analysis

**Figure 3:**
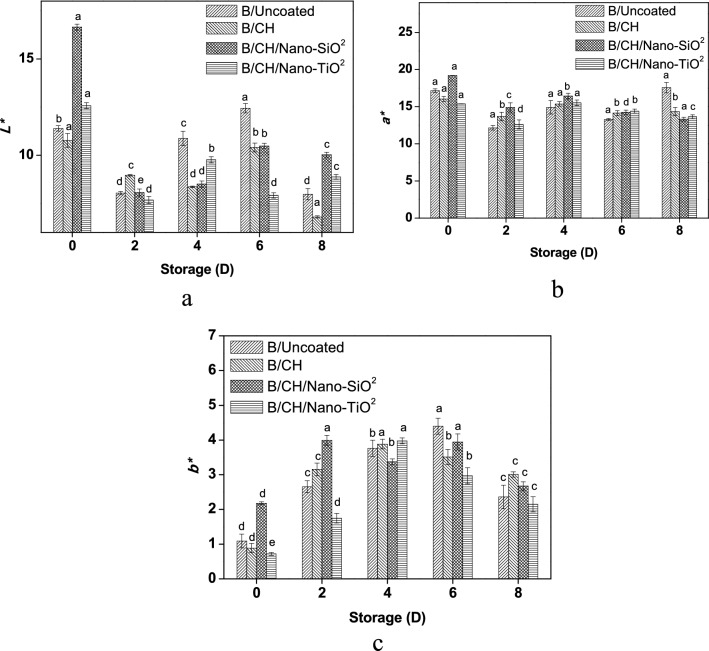
Effects of coating films on colour index L* (**a**), a* (**b**) and b* (**c**).

In terms of the colorimetric coordinates during the whole storage. Though, on the 6th day, lightness values were similar to each other compared with (B/Uncoated), Fig. 3a. Enzymatic processes and weight loss could be the main reasons for the visual quality reduction. (B/CH) coating maintained lightness (6.80% loss) as compared to other nano-coatings, whereas (B/Uncoated) samples reported the maximum (7.98% loss).

(a*) values were decreased until the second day of the whole storage period then began to increase, Fig. 3b. Both (B/CH/Nano-SiO_2_) followed by (B/CH/Nano-TiO_2_) coated blueberry samples reported lower values against the (B/Uncoated), (13.31%, 13.65–17.57% loss) at the end of the storage period, respectively.

The epidermis of blueberries was characterized such as violet, though differences among various coating films treatment occurred during 8 days. (B/CH/Nano-TiO_2_) coating treatment (2.97% loss) exhibited lower b* values beginning from the 6th day against the (B/Uncoated) samples (4.40% loss), Fig. 3c.

The result reported that various coating films and storage time had effected on the firmness of blueberry samples, which decreased with the increasing time, Table 1. By 8th day, (B/CH/Nano-TiO_2_) coating film retained the greatest firmness (9.03 N m^−1^ loss); whereas, (B/Uncoated) samples recorded the lowest firmness (18.09 N m^−1^ loss). Mannozzi et al.^1^, established similar firmness results of coated blueberries samples due to the berry skin inflexibility.

**Table 1:** Effects of coating treatments on firmness.

Storage (days)	Firmness (N m^−1^)			
	B/uncoated	B/CH	B/CH/nano-SiO_2_	B/CH/nano-TiO_2_
0	3.80 ± 0.20^b^	8.31 ± 0.29^ab^	7.95 ± 0.55^a^	3.37 ± 0.28^a^
2	4.17 ± 0.20^c^	9.18 ± 0.29^bc^	9.26 ± 0.55^a^	6.92 ± 0.29^a^
4	6.00 ± 0.41^d^	11.00 ± 0.07^c^	11.62 ± 0.44^a^	8.23 ± 0.07^ab^
6	10.22 ± 0.59^d^	14.77 ± 0.34^bc^	13.21 ± 0.54^a^	8.40 ± 0.34^b^
8	18.09 ± 0.16^a^	17.72 ± 0.53^a^	15.44 ± 0.88^a^	9.03 ± 0.53^a^

*Values within a column (lowercase) are significantly different (*p* ≥ 0.05). The values in parentheses indicate (SD ±) standard deviation.

### Phytochemical changes

**Figure 4:**
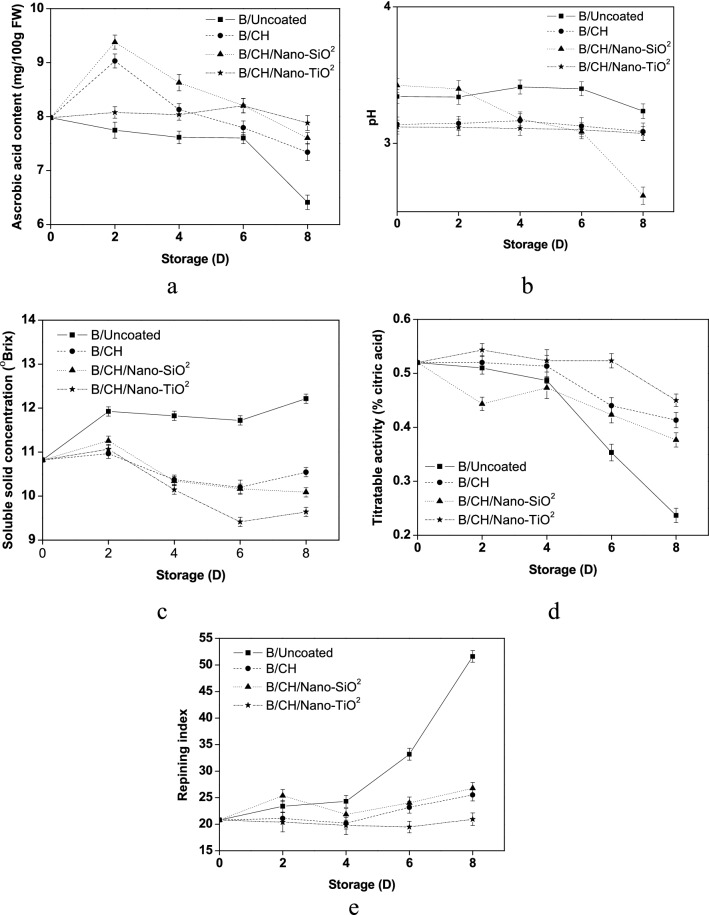
Phytochemical changes of ascorbic acid content (**a**), pH (**b**), soluble solid concentration (**c**), titratable acidity (**d**), and repining index (**e**) for coated blueberry samples.

Various coating films preserved the ascorbic acid content as shown in (Fig. 4a). On the 8th day, (B/Uncoated) established the lowest values (6.41 g/100 g FW) as compared to the coated blueberries due to the ascorbic acid spontaneous oxidation. (B/CH) reported the highest content (7.34 g/100 g FW) followed by (B/CH/Nano-SiO_2_) (7.60 g/100 g FW), while (B/CH/Nano-TiO_2_) recorded a little lower ascorbic acid value (7.88 g/100 g FW). Similarly, nanocoatings based on chitosan have reported a delay of ascorbic acid contents in treated blueberries^9^.

The pH values for (B/Uncoated) blueberries were slightly raised from 3.34 to 3.40 on the 6th day then decrease to 3.24 at the end of the storage time, (Fig. 4b). (CH/Nano-SiO_2_) coating films might report a good-quality fortification for pH value.

The effects of nano-material film coatings on soluble solid concentrations are shown in Fig. 4c. Soluble solid concentrations were increased during the whole storage time and ranged from 10.82 to 12.21% for (B/Uncoated) blueberry fruits and from 10.82 to 9.64% for coated blueberry fruits. Titratable acidity values for (B/Uncoated and coated blueberries) were found to be around (0.52–0.24% citric acid) and (0.52–0.38% citric acid), respectively, Fig. 4d. (B/CH/Nano-TiO_2_) film established the highest titratable acidity values (0.45% citric acid).

Coated blueberries retained the ripening index (SSC/TAA ratio) obviously as shown in Fig. 4e. (B/Uncoated) samples recorded minimum increment between the 2nd day (23.38%) to the 6th day (33.17%) then the highest increment until the 8th day (51.61%) as compared to the other coated berries. (B/CH/Nano-TiO_2_) reported the lowest ripening ratio (20.94%) followed by (B/CH) (25.51%), while (B/CH/Nano-SiO_2_) recorded a little high percentage (26.78%).

### Oxidation enzymes and anthocyanin contents

**Figure 5:**
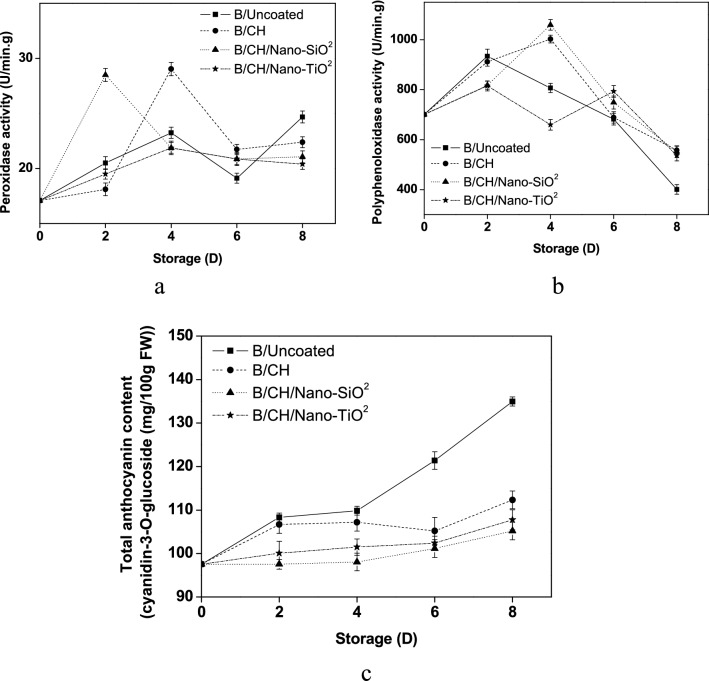
Effects of coating treatments on oxidation enzymes (**a**,**b**) and anthocyanin contents (**c**).

According to Fig. 5a, the results established high inactivation of peroxidase (POD) enzyme activities by (B/CH/Nano-TiO_2_) (20.39 U/min g) whereas (B/CH/Nano-SiO_2_) (21.06 U/min g) reported a little higher compared to (B/Uncoated) (24.67 U/min g) samples.

The effect of various coating films on polyphenoloxidase (PPO) enzyme activity is shown in Fig. 5b. During 0–4 days of storage at commercial temperature, all blueberry treatments except (B/CH/Nano-TiO_2_) (659.45 U/min g) reported a gradual increase in (PPO) activity. A parallel finding was established on ready-to-eat cantaloupes stored in a chilling condition that nano-coatings films may limit the O_2_ concentration to explain the process of oxidation–reduction^9^. The results at the end of the storage period demonstrated the successful coating treatment was (B/CH) (558.03 U/min g) followed by (B/CH/Nano-SiO_2_) (551.63 U/min g).

The total anthocyanins in (B/Uncoated) samples increased compared to the other coated blueberries during the storage time passage. By the 8th day, (B/CH/Nano-SiO_2_) samples established the slightest change in (TAC) (105.19 cyanidin-3-*O*-glucoside mg/100 g FW) compared to (B/CH/Nano-TiO_2_) and (B/CH) samples (107.76 and 112.34 cyanidin-3-*O*-glucoside mg/100 g FW), respectively, Fig. 5c.

### Microbial population measurements

**Figure 6:**
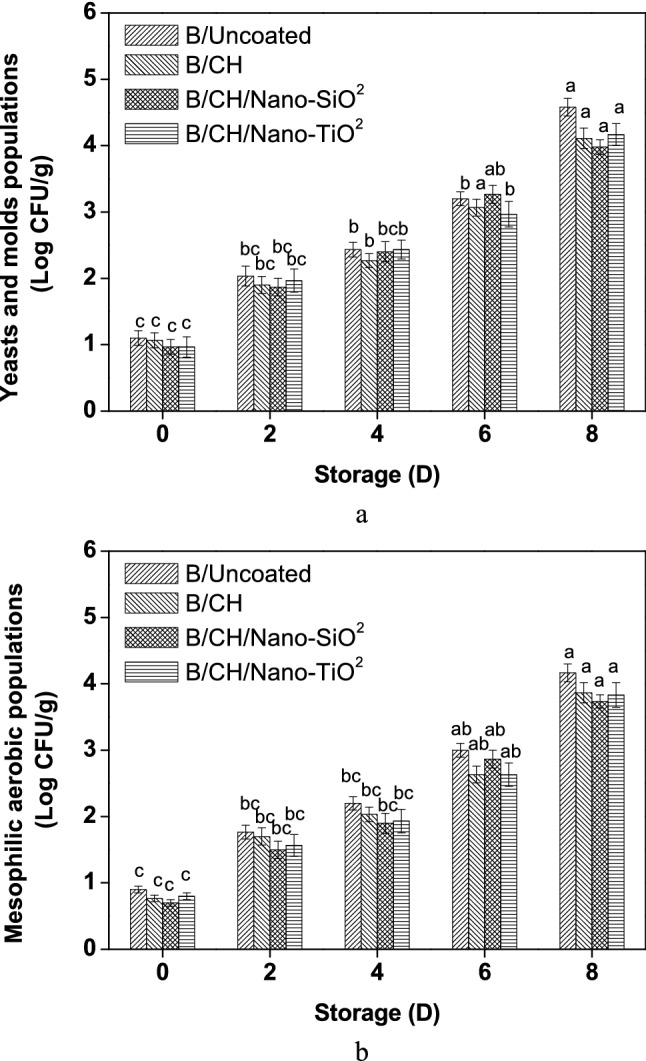
Microbial population measurements on coated on blueberries.

Results on yeasts and molds populations of various coatings are shown in Fig. 6a. Blueberry samples treated (B/CH/Nano-SiO_2_) minimized the growth to values < 4 log CFU/g against (B/Uncoated) and other coating films that might be associated with fungicide action of the (Nano-SiO_2_)^11^. (B/CH) established (4.11 log CFU/g) when compared to (B/Uncoated) (4.85 log CFU/g) during 8 days of storage due to the efficiency of antifungal activity of the chitosan component^14^.

As shown in Fig. 6b, mesophilic aerobic population reductions were effectively established for (B/CH/Nano-SiO_2_) film as (3.73 log CFU/g). Moreover, (B/CH/Nano-TiO_2_) and (B/CH) recorded similar values (3.83 and 3.87 log CFU/g), respectively. In agreement with the population results, Alvarez et al., (30) established the strong effect of chitosan (2%) on the mesophilic aerobic population of blueberry samples.

## Discussion

Chitosan alone can control the fungus growth as Anthracnose (*Colletotrichumacutatum*), Alternaria (*Alternaria *spp.), and *Botrytis *(*Botrytis cinerea*)*.* Chitosan can penetrate inside the fungus nuclei, cell membranes proteinaceous and intracellular constituents interferes that leads to its inhibition^7^. Moreover, nano-coatings developed blueberries shelf life by forming a physical barrier for respiration, dehydration, and senescence^10,15^.

Colour is a vital indicator of a blueberry sample's quality. The lightness loss can be due to the presence of nano-film coatings which can cause some surface changes^9^. The polyphenol oxidation reactions can be a reason for the colour decline^16^. Blueberries lose firmness during the postharvest storage, that leads to shelf-life limitation. Nano-coating with chitosan combination films reduces softening rates by reducing the metabolic activity as it performs as O_2_ and CO_2_ uptake barriers^17^.

Nano-coating with chitosan combination films might conserve the ascorbic acid contents against (B/Uncoated) samples due to the oxidation–reduction on the permeable surface and exchange gas limitation^18^. Karolina et al.,^13^ reported that the ascorbic acid reduction may be linked with the oxygen reduced diffusion and low respiration rate. The pH values decreases are in charge of fruit preservation against fungal and bacterial growth by using edible coating films^3^. The soluble solid concentration of (B/Uncoated) blueberries were higher than those of coated samples due to the maturation and over ripening. The increase of soluble solid concentration is probably associated with the moisture loss, as blueberries were harvested fully ripe^19^. Karolina et al.^13^, reported that coatings may change the atmosphere around fruits, that as a result can reduce metabolism rates. Citric acid is the major organic acid in the blueberries juice that can refer to the fruit ripens, while the lowest value for (B/Uncoated) samples was due to the fungal population^20^. In a word, titratable acidity values were positively influenced by delaying maturation with the presence of coating films during the storage period. These results could report that the nano-material and chitosan combinations is acceptable for the gradual maturation^3^. Repining index is a vital *quality* parameter which evaluates the acidity and sweetness of fruits. Coating films especially with chitosan might create a modification of the interior atmosphere in coated several fruits. The performance was justified to the correlation between soluble solid concentration/titratable acidity ratio as any reductions in titratable acidity values would produce increases in the repining index, that will result in a sugary taste to the treated samples^21^. Consequently, nano-materials were effective in blueberry ripening delay.

Peroxidase enzyme activity (POD and PPO) is essential in diphenols reduction. The efficiency of the activity results depends on quite a lot of factors such as the ripening stage, storage, treatment and duration applied^22^. Rok^9^, established that nano-coating materials may clarify the oxidation–reduction due to the oxygen rate decrease. Guohua et al.^10^, established that (POD and PPO) activity reductions might be due to blueberries abiotic stress. Anthocyanin pigments are in charge of the blue and red colour in the horticultural and any changes in values could be due to the phenolics catalyzed oxidation by (POD,PPO) and continuous biosynthesis during the storage^6,23^.

The blueberry's quality is influenced by microorganisms such as yeasts and fungal infections. The combination of (chitosan + Nano-SiO_2_) can result in enhancing the antifungal capacity and limit the microbial population to extend the fresh blueberry shelf life at commercial temperature.

As a result, the optical estimation confirmed that (B/Uncoated) fruits had widespread microbial growth on the surface at the end of the storage period. The chitosan-coating film with or even without nano-materials inhibited the microbial population.

In conclusions, composite nano-films are eco-friendly, performed with chitosan have been enhanced physical, mechanical, phytochemical, and nutritional components with microbial population inhibition on fresh blueberry fruits during commercial storage. The results established that using nano-material coating films can extend the shelf-life, delay ripening, and control spoilage such as yeasts, mould populations, and mesophilic aerobic counts. Blueberry fruits influenced by chitosan film alone achieved better lightness for colour index. Formation semi-films by nano-materials especially with (SiO_2_–TiO_2_) is needed for nanotechnology application and preservation industries for consumable vegetables and fruits.

## Materials and methods

### Materials and fruit sample

Chitosan (85%), nano-(silicon/titanium dioxides 15 nm) and glycerol, acetic acid were from (Sigma, USA). Four Kg of fresh blueberries (*Vacciniumashei *L.) in relative humidity (80–90%) were procured from a local grocery store, transported to the laboratory of the college of science, Taif University.

### Coating films preparations

Chitosan film 1% (B/CH) was arranged by blending acetic acid and glycerol. The coating film was stirred over night at 300 rpm and centrifuged at 4 °C for 30 min. Chitosan-nano-silicon dioxide (B/CH/Nano-SiO_2_) and chitosan-nano-titanium dioxide (B/CH/Nano-TiO_2_) films were arranged by adding 1% of Nano-SiO_2_ and TiO_2_ to the chitosan solution, respectively. While, (B/Uncoated) was the treated samples with deionized water.

### Fruit treatments and storage

The uniform blueberries were selected in size, colour, weight, relative humidity in-between 80 and 90%, and without any sign of optical physical damage or even microbial contaminations from an orchard in Harbin, Heilongjiang Province, China. The bulk was divided into four quarters according to the experimental design and stored into holy small boxes. Blueberries were dipped into various coating films, dried, and stored at the commercial temperature (27 ± 0.5 °C) for up to (8 days) and analyzed on days 2, 4, 6, and 8 for all the postharvest quality parameters.

### Physical and mechanical parameters

Each blueberry group was regularly weighed using a scale with an accuracy of (0.01 g) and calculated as accumulated weight loss and decay rate as a percentage per unit storage time, Eqs. (1) and (2)^13^.

1 $$Weight\, loss \left(\%\right)=\left(1-\frac{{w}_{f}}{{w}_{i}}\right)\times 100$$

where ${w}_{i}$ is the initial blueberry weight and ${w}_{f}$ is the blueberry weight during the storage.

2 $$Decay\, Rate \left(\%\right)=\left(1-\frac{{V}_{t}}{{V}_{o}}\right)\times 100$$

where ${V}_{t}$ is the blueberry apparent volume at a certain degree of dryness after time $t$ and ${V}_{o}$ is the raw blueberry apparent volume.

Blueberries colour index such as (L*, a*, and b*) were evaluated by using (CR-400 Chroma Meter, USA) with an 8 mm aperture^24^. Firmness texture analysis for various coating treatments was achieved by using (TAXT Plus, UK) with 13 mm probe height, a distance of 3 mm, 50 kg load transducer and pretest speed of (2 mm/s) and test speeds of (1 mm/s) with an automatic trigger (5 g)^10^. Firmness was expressed as newton per meter (N m^−1^) by determination of fifteen pieces per replicate.

### Determination of phytochemical contents

The various coated blueberries for all treatments (50 g) were blended for the determination of phytochemical contents. Ascorbic acid content was detected by the oxalic acid titration method explained by Rok and expressed as (mg/100 g FW)^9^. pH measurements are essential indicators for the microbial growth which might cause the spoilage. The pH values were examined using a pH meter (320, Toledo, China). After berry juice homogenization, pH readings were evaluated by the direct electrode inundation^3^. Juice from the blueberry samples was used for soluble solid concentration (°Brix) by using a refractometer (Model 30PX, USA). Titratable acidity was detected according to 942.15 AOAC methods^25^, by determining 0.1 mol L^−1^ sodium hydroxide until pH equals (8.1). Results were calculated as a percent (grams of citric acid equivalent/100 g), Eq. (3).

3 $$Titratable\, Acidity \left(\%\right)=\frac{0.1\, \mathrm{N\, NaoH}\, used \times 0.0064}{ml\, of\, juice\, used}\times 100$$

The Repining index was applied by the quotient between both titratable acidity (TAA) and soluble solid concentration (TSS), Eq. (4)^10^.

4 $$Ripening\, Index =\frac{TSS}{TAA}$$

### Determination of peroxidase and polyphenoloxidase enzymes and anthocyanin contents

Approximately 5 g of coated blueberry samples were weighed, homogenized in 100 mL of sodium phosphate buffer (100 mM, pH 7.0), centrifuged at 16,000 r/min, filtered, and stored at -18 °C for polyphenoloxidase enzyme determination. 2.2 mol/L sodium phosphate buffer, and (0.5 mL) of 0.175 mol/L catechol solution were added to gain the ionically bound enzymes for the extracts and detected at (410 nm)^11^. Peroxidase enzyme was determined by blending 5 g of coated blueberries with (3 mL) 0.1 mol/L pyrocatechol solution, centrifuged at 16,000 r/min, and filtrated with a detection at (460 nm)^26^. Total anthocyanin contents (TAC) in blueberry samples were evaluated by using a spectrum spectrophotometer (754 PC, China) at (520 nm). Results were expressed as cyanidin-3-*O*-glucoside equivalents (mg/100 g FW)^27^.

### Microbial population studies

Blueberries microbial populations were examined by placing 15 g of various coatedsamples in a sterile plastic bag with 85 mL of the peptone water and were homogenized by using a Stomacher (400 Circulator, United Kingdom). Serial dilutions (10^−1^, 10^−2^, and 10^−3^) were made until the surface spreading on the selective media (3 M 212 petrifilms)^28^. Mesophilic aerobic populations were incubated at 37 °C for 2 days, while yeasts and molds populations were incubated at 25 °C for 5 days. Results were calculated as log CFU/g in three sample containers with three replicate counts of each treatment.

### Statistical analysis

All date results were expressed as (mean ± SD) with three replicates for each value and analyzed by SPSS (26.00 software), while the mean separation was significantly (*p* ≤ 0.05) by the Duncan's multiple tests.
